# Hepatic Artery Mycotic Aneurysm Associated with Staphylococcal Endocarditis with Successful Treatment: Case Report with Review of the Literature

**DOI:** 10.1155/2013/610818

**Published:** 2013-05-12

**Authors:** Dhara Chaudhari, Atif Saleem, Pranav Patel, Sara Khan, Mark Young, Gene LeSage

**Affiliations:** ^1^Department of Internal Medicine, East Tennessee State University, Quillen College of Medicine, 1008 Quality Circle, Apartment 79, Johnson City, TN 37615, USA; ^2^Department of Gastroenterology, East Tennessee State University, Quillen College of Medicine, 1008 Quality Circle, Apartment 79, Johnson City, TN 37615, USA

## Abstract

Mycotic hepatic artery aneurysm is a vascular pathology associated with bacterial endocarditis. It is rare in occurrence after the introduction of effective antibiotics. We present a young patient with injection drug abuse associated staphylococcal endocarditis which was successfully treated with antibiotics and valve replacement who presented with abdominal pain. He was found to have mycotic aneurysm of hepatic artery which was successfully treated with coil embolization.

## 1. Introduction 

Hepatic artery aneurysm is an uncommon vascular lesion. Mycotic hepatic artery aneurysm is rare but recognized complication of bacterial endocarditis. The incidence of mycotic aneurysm has decreased following the widespread use of effective antibiotics. We present a case of hepatic mycotic aneurysm in a patient with staphylococcal bacterial endocarditis.

## 2. Case

**Figure 1:**
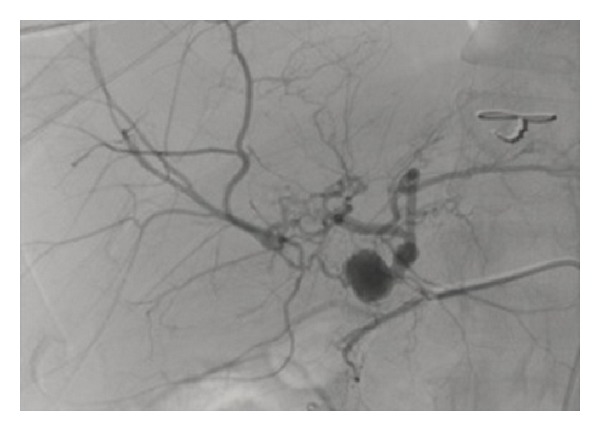
Hepatic angiography showing right hepatic artery aneurysm (1.2 × 1.0 cm) and left hepatic artery aneurysm (5 × 6 mm).

**Figure 2:**
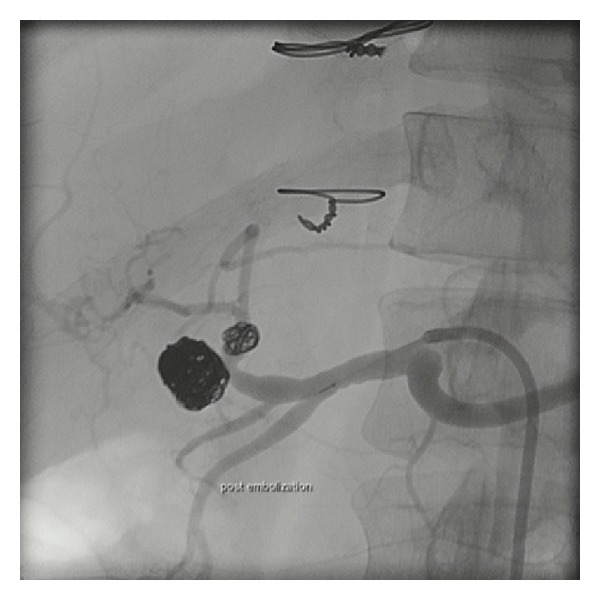
Postembolisation celiac angiography showing successful coiling of both aneurysms.

 A 27-year-old male presented with complain of chest pain and abdominal pain. Patient had recent history of Methicillin Resistant *Staphylococcus aureus* (MRSA) aortic valve endocarditis and septic emboli to coronary arteries requiring double vessel bypass grafting with aortic valve replacement 5 months ago. He also had history of hepatitis C. He denied dyspnea, orthopnea, paroxysmal nocturnal dyspnea, fever, or chills. He also denied melena, hematochezia, or change in bowel habits. He had previous history of smoking and self-injecting drug abuse. Family history and review of system were unremarkable. Physical exam was significant for systolic heart murmur 3/6 in intensity, otherwise unremarkable systemic examination. His home medicines included aspirin, carvedilol, plavix, lisinopril, and sertraline. Laboratory data revealed white blood cell count (WBC) 10.5/L, potassium (K) 3.1 meq/l, total bilirubin 0.5 mg/dL, aspartate transaminase/alanine transaminase 12/10 IU/L, and alkaline phosphate level 75 U/L; urine drug screen positive for cannabinoids; chest X ray: no acute lung process; electrocardiogram (EKG): no acute ST-T changes. The patient was admitted to rule out acute coronary event, and infectious disease was consulted for further treatment recommendation. Computed tomography (CT) abdomen/pelvis was ordered for unexplained abdominal pain which revealed possible hepatic pseudo-aneurysm and CT Abdomen/pelvis angiography revealed right hepatic artery aneurysm of 1.2 × 1.0 cm size with no flow distal to aneurysm and left hepatic artery aneurysm of 5 × 6 mm size (Figure 1). Patient underwent coiling and successful embolization with nine coils in right hepatic artery aneurysm and four coils in left hepatic artery aneurysm (Figure 2).

## 3. Discussion 

Mycotic aneurysm is defined as an infectious break in the wall of an artery with formation of a blind, secular outpouching, that is, contiguous with the arterial lumen. The common sites of involvement in descending order are aorta, peripheral artery, cerebral artery, and visceral arteries [1]. Among the visceral arteries, superior mesenteric artery and arteries in liver, spleen, kidneys, or lungs has been reported in the literature [2]. Mycotic hepatic artery aneurysm is a rare but important vascular pathology accounting for 0.1% of all arterial aneurysms and 20% of all visceral aneurysms [3]. Mycotic aneurysm was first described by Osler in 1885. The term “mycotic” is a misnomer as fungal infections are uncommonly associated with this pathology but was used as it looks like fungal growth due to beaded appearance and multiple aneurysms [4]. 

In preantibiotic era, most of hepatic artery aneurysms (HAA) were mycotic and associated with bacterial endocarditis [5]. Most common causative pathogens are *Streptococcus* and *Staphylococcus* groups. There are few cases reported in the literature of mycotic hepatic artery aneurysm with staphylococcal endocarditis [5–9]. Also methicillin resistant strain of staphylococcus is more pronounced in intravenous drug users. Our patient had staphylococcus endocarditis secondary to injection drug abuse which we believe was the primary risk factor leading to formation of the mycotic aneurysm. In addition, Gram-negative bacteria, fungal infection with *Aspergillus* or *Candida,* and *Mycobacteria* are rare causes of mycotic aneurysm [1]. 

After introduction of antibiotics, incidence of mycotic aneurysm has decreased [5]. Currently, most of the hepatic artery aneurysms are primary, likely due to medial degeneration or secondary atherosclerosis. Less commonly, polyarteritis nodosa, trauma, fibromuscular dysplasia, and infectious pathology like acute pancreatitis or cholecystitis have been reported in the literature [10]. Immunocompromised states such as immunodeficiency, diabetes, or malignancy can also predispose to mycotic aneurysm [1]. Although rare, hepatic artery aneurysms have also been reported after liver transplantation [11]. Stengal and Wolferth in 1923 reported 217 patients with mycotic aneurysm, of which 187 had endocarditis and 19 patients had hepatic artery aneurysm [12]. This contrasts with a more recent study from the Mayo clinic, which documented 306 patients with true visceral aneurysm diagnosed between 1980 and 1999 with 36 patients (12%) with hepatic artery aneurysm and only one patient with hepatic mycotic aneurysm [13]. 

The pathogenesis of mycotic aneurysm can be explained by lodging of infected emboli into lumen of normal vessel leading to inflammation and necrosis, followed by weakening of the arterial wall causing dilation [14]. Other elucidated etiologies could be circulating organisms targeting preexisting vascular defect or contiguous vessel involved from adjacent infectious source [1, 14]. Hepatic artery aneurysm may involve intrahepatic (25%), extrahepatic (75%), or common hepatic artery. The common hepatic artery is the most frequent site (63%), followed by the right hepatic artery (28%), the left hepatic artery (5%), and both hepatic arteries (4%) [15]. In our case, aneurysms were in both right and left hepatic arteries. 

Although most hepatic artery aneurysms are asymptomatic [13], when symptomatic 80% of patients present with right upper quadrant pain. Gastrointestinal hemorrhage [16] and jaundice [9] can be associated with the abdominal pain. The triad of epigastric pain, hemobilia, and obstructive jaundice suggestive of Quincke's symptom are seen in one-third of patients [17, 18].

Hepatic artery aneurysm has high risk of rupture especially mycotic aneurysm because of dislodgment of infected emboli causing vessel wall inflammation and necrosis. It may rupture directly into the intestinal tract presenting as either upper or lower gastrointestinal bleeding or can rupture into peritoneal cavity presenting with symptoms of peritonitis [16, 17]. When it ruptures into adjacent portal or hepatic venous system, the patient can present with jaundice. 

The widespread use of ultrasonography especially color doppler ultrasonography or CT scan has resulted in increased identification of asymptomatic hepatic artery aneurysms [8, 10]. A multidetector CT scan can provide three-dimensional anatomy of the visceral arteries [19]. MR angiography is an alternative for patients who cannot have CT angiography. The gold standard test is conventional angiography which provides accurate location, size, and shape of the aneurysm. There have been sporadic cases reported of PET scan detecting arterial aneurysms [1]. 

Treatment of hepatic artery mycotic aneurysm depends on size, location/anatomy, and comorbidity. Treatment is recommended in symptomatic patients who have a higher risk of rupture and aneurysm with a diameter greater than 2 cm. Treatment modalities are embolization with coils or endovascular stenting and surgery with or without revascularisation [20]. Embolization with coils is the treatment of choice for intrahepatic lesion, high-risk surgical candidates, secular aneurysm, and proximal aneurysm. Surgical options include ligation, ligation excision, and hepatic lobe resection. Surgical options are suitable to patients with low risk and usually distal aneurysm [10]. 

## 4. Conclusion 

Mycotic hepatic aneurysm is a rare entity in antibiotic era. Because of their asymptomatic nature, high index of suspicion is required for diagnosis. The patients with recent or remote history of endocarditis or sepsis are at risk. Mycotic aneurysm, if not treated, is associated with high-risk rupture and increased mortality.
